# Stop stunting: situation and way forward to improve maternal, child and adolescent nutrition in Afghanistan†

**DOI:** 10.1111/mcn.12288

**Published:** 2016-05-17

**Authors:** Ariel Higgins‐Steele, Piyali Mustaphi, Sherin Varkey, Humayoun Ludin, Najibullah Safi, Zulfiqar A. Bhutta

**Affiliations:** ^1^ UNICEF Afghanistan Country Office UNOCA Jalalabad Road Kabul Afghanistan; ^2^ Ministry of Public Health Kabul Afghanistan; ^3^ Center of Excellence in Women and Child Health The Aga Khan University Karachi Pakistan; ^4^ Centre for Global Child Health Hospital for Sick Children Toronto Canada

While Afghanistan has made progress in improving the nutritional status of children and women, rates of under‐nutrition, stunting and wasting remain among the highest in the world, requiring attention to address immediate and underlying causes (Varkey *et al*. 2015). Given potential human, societal and economic gains from investment in nutrition (Branca *et al*. 2015) reducing the dual burden of acute and chronic undernutrition must figure among social sector and development priorities for the country.

Comparisons of two national nutrition surveys in Afghanistan (2004 and 2013) show gradual improvements in the nutritional status of women and children, and yet, the rates of undernutrition continue to be too high. According to a National Nutrition Survey in Afghanistan in 2004, the prevalence of chronic malnutrition (stunted linear growth or low height‐for‐age) among children aged 6 to 59 months was 60.5%; in the same age group, the prevalence of acute malnutrition (wasting) was 8.7% (Afghanistan Ministry of Public Health, National Nutrition Survey 2004). The 2013 survey showed malnutrition rates among children 0 to 59 months of age with stunting at 40.9%, severe stunting at 20.9%, and moderate stunting at 20% (Afghanistan Ministry of Public Health, National Nutrition Survey 2013). Wasting or acute malnutrition, however, increased slightly from the previous survey to 9.5%, with moderate acute malnutrition at 5.5% and severe acute malnutrition at 4.0% (Afghanistan Ministry of Public Health, National Nutrition Survey 2013). These findings must be interpreted with caution given that the 2004 survey sample was much smaller than the 2013 survey. For the first time, child obesity was measured, and it was found that 5.4% of the children aged 0–59 months were overweight (Fig. 1).

**Figure 1:**
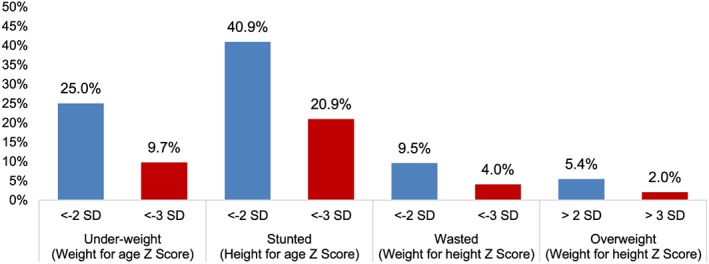
Nutritional status of children aged 0–59 months in Afghanistan (National Nutrition Survey, 2013).

The recent survey also reports moderate improvement in undernutrition in women of reproductive age in Afghanistan, although maternal wasting and micronutrient deficiencies remain widespread. Among women of reproductive age (15–49 years), 9.2% of women are thin or undernourished (BMI < 18.5 kg/m2). The proportion of women with mild thinness is 6.7% and severe thinness is 2.4%. The nutritional status of adolescent girls (aged 10–19 years) was assessed for the first time in Afghanistan in the 2013 survey, showing that 8% of adolescent girls are thin and 1.5% are severely thin. Anaemia (Hb levels < 11.99 gm/dl) is common in women of reproductive age (40.4%), among children 6–59 months of age (44.9%), and adolescent girls of 10–19 years (29.9%) (Afghanistan Ministry of Public Health, National Nutrition Survey 2013). Given that up to a fifth of all stunting in young infants may be associated with fetal growth retardation (Black *et al*. 2013) and the recognized association of stunting with maternal height in neighbouring Pakistan (Di Cesare *et al*. 2015), the relationship of adolescent and maternal nutrition with childhood stunting should be considered in the context of the mother–infant dyad and intergenerational effects of interventions.

Equity is a central issue, and major disparities in nutritional status in Afghanistan exist across geographic locations and socio‐economic groups. The prevalence of acute undernutrition is higher in several provinces: Uruzgan (22%), Nangahar (21%), Nuristan (19%), Khost (18%), Kandahar (14%) and Helmand (14%). Higher prevalence of stunting was shown in Farah (70%), Nuristan (63%), Kunar (56%), Paktia (55%) and Nangahar (52%) (Afghanistan Ministry of Public Health, National Nutrition Survey 2013) (Fig. 2).

**Figure 2:**
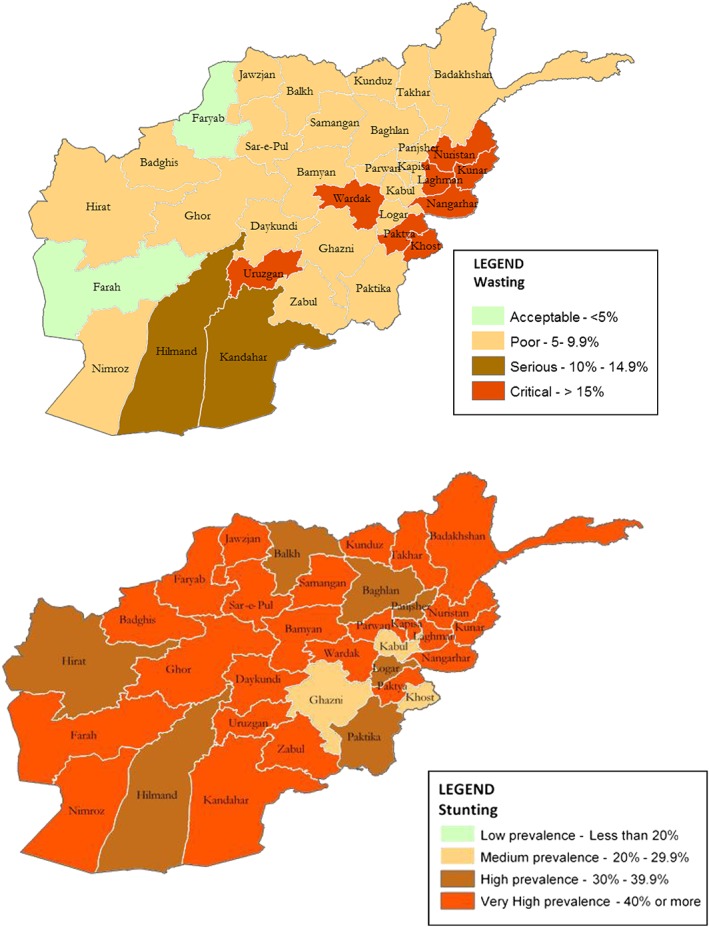
Distribution of stunting and wasting by province in Afghanistan (National Nutrition Survey, 2013).

About 30.7% of Afghan children under 5 years in the poorest households are more likely to be underweight compared with 17.7% of children from the richest households. For acute malnutrition, about 9.8% of the children under 5 years of age in the poorest households are more likely to be wasted (low weight‐for‐height) compared with 6.8% in the richest households. About 49.4% of the children under 5 years of age in the poorest households are more likely to be stunted (low height‐for‐age) compared with 31.1% in the richest households (Afghanistan Ministry of Public Health, National Nutrition Survey 2013). There are other measures of inequity as well and significant differentials are also noted by geography, gender, parental education – especially maternal – and ethnicity.

Although the high poverty rate in Afghanistan influences these rates, many other determinants contribute to undernutrition of children and women, including those related to health status, dietary intake, food availability, care of mothers and children, health environment and services, and public policies and laws. Poor sanitation and hygiene lead to higher rates of childhood illnesses, including diarrhoea and pneumonia, and could well contribute to chronic enteropathy and associated linear growth retardation and stunting. Dietary intake is suboptimal, with only 14.2% of children aged 6–23 months from the poorest households likely to get a minimum acceptable diversified diet and about 31% in the richest households (Afghanistan Ministry of Public Health 2013). Poor hygiene, sanitation and limited safe water supply are other major causes of infectious illnesses, and just over half (56.7%) of the population has access to improved drinking water sources (Central Statistics Organisation (CSO) & UNICEF, 2012).

Maternal, newborn and child care practices are also suboptimal. Less than half of women give birth in a health facility with a skilled birth attendant (Central Statistics Organisation (CSO) & UNICEF, 2012; Black *et al*. 2013). While there has been steady improvement in the prevalence of children aged 0–6 months who are exclusively breastfed in Afghanistan, the rate of 58% means almost half of infants are not exclusively breastfeed (Afghanistan Ministry of Public Health, National Nutrition Survey 2013) (Fig. 3).

**Figure 3:**
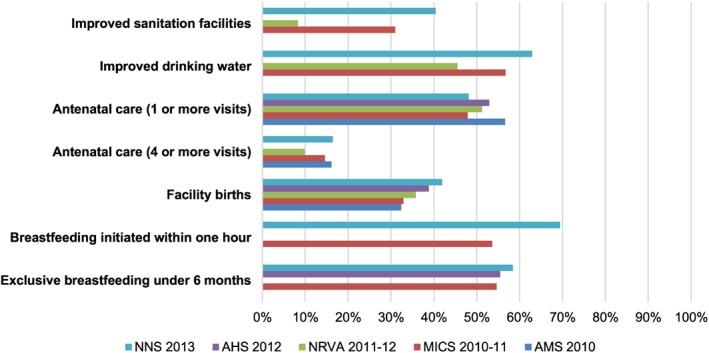
Selected indicators affecting nutritional status of children in Afghanistan from recent national surveys.

Showing high‐level political commitment to improve maternal, child and adolescent health, the Afghan government has made new commitments in 2015 as part of *A Promised Renewed* and *Call to Action for Maternal and Child Survival*, through its Kabul Declaration, and the *Global Strategy for Women's*, *Children's and Adolescents' Health (2015‐30)*, including to reduce the rate of stunting in children under 5 years of age to 30% by 2020 and 10% by 2030. With the government's strong political will to immediately adhere to this new global strategy with evidence‐based targets, progress can be made through further inputs into supply, demand and quality dimensions.

To achieve these new targets, the Ministry of Public Health is revising the National Nutrition Strategy with a detailed costed plan that advocates for increasing investments for nutrition. There is a clear need to scale‐up both nutrition‐specific and ‐sensitive programmes in Afghanistan, given findings from the recent survey reveal a double burden of undernutrition – stunting and wasting – among children under 5 years of age.

The Ministry of Public Health, with support from its development partners, is prioritizing the following areas with a focus on the first 1000 days of life (Bhutta *et al*. 2013):

## Nutrition‐specific interventions

- Infant and young child feeding promotion programmes, including support for exclusive breastfeeding and complementary feeding both at the facility and community levels, and promotion of dietary diversity;
- Maternal nutrition interventions, including micronutrient supplementation, nutrition counselling and behaviour change communication, food fortification including iodised salt.
- Adolescent girls' nutrition interventions, including weekly iron folic acid supplementation for both in‐school and out‐of‐school adolescent girls;
- Micronutrient supplementation and fortification programme; and
- Treatment of acute malnutrition.

## Nutrition‐sensitive interventions

- Food and nutrition situational monitoring, assessments and surveillance;
- Creating linkages with livelihoods/income generating programmes to improve asset base of households;
- Improving water, sanitation and hygiene;
- Building the capacity of partners and government to deliver quality programmes; and
- Use of appropriate programme delivery platforms for nutrition specific interventions:
  - communities for nutrition education and promotion;
  - health facilities especially through integrated management of childhood illness (IMCI);
  - private sector ‐ large‐scale food fortification;
  - school‐based delivery platforms for adolescent girls; and
  - social protection programmes including cash transfers.

Appreciating the multisectoral nature of undernutrition, there is also a need to create a forum at the highest level of government including the sectors of health, agriculture, education, finance, economy, rural development and public works to drive collective and integrated action for nutrition.

Over the past decade, Afghanistan has shown that progress in improving nutritional status of children and women is possible, even in challenging economic, security and political circumstances. New commitments for reductions in stunting and increased coverage of evidence‐based interventions can be realized through multi‐sectoral collaboration, led by the Ministry of Public Health and supported by its development partners. Nutrition must continue to be positioned as a central public health issue in Afghanistan with profound effects and potential for social and economic development.

## Source of funding

None.

## Conflicts of interest

The authors declare that they have no conflicts of interest.

## Contributions

AHS developed the drafts for the Commentary. PM and SV reviewed the drafts and provided technical inputs for the commentary. HL and NS provided inputs based on their involvement in the development, implementation, and analysis of the National Nutrition Survey (2013). ZAB was the principal investigator for the NNS 2013 and has provided technical assistance in data analysis and interpretation. All authors read and approved the final version.
